# Cure of Habitual Constipation by Intra-Abdominal Use of Mineral Oil

**Published:** 1914-09

**Authors:** 


**Cure of Habitual Constipation by Intra-Abdominal Use of Mineral Oil.** W. F. Burrows, Med. Rec., April 11, 1914, advises, after breaking up adhesions, correcting angulations, ptosis, etc., the pouring of 8 or 10 ounces of pure liquid petrolatum of high specific gravity into the abdominal cavity.

